# Treatment of bilateral sacral insufficiency fractures with sacroplasty in a patient with motor complete tetraplegia

**DOI:** 10.1038/s41394-021-00393-7

**Published:** 2021-03-19

**Authors:** Marla A. Petriello, Suzanne Groah, Suman D. Matiana

**Affiliations:** 1 MedStar Georgetown University Hospital, Department of Rehabilitation Medicine, Washington, DC USA; 2 MedStar National Rehabilitation Hospital, Washington, DC USA; 3 Inova Alexandria Hospital, Department of Medicine, Alexandria, VA USA

**Keywords:** Pain, Spinal cord diseases

## Abstract

**Introduction:**

Osteoporosis is a common secondary complication of spinal cord injury, with fragility fractures typically occurring in the lower body and management often is conservative.

**Case presentation:**

We present a 52-year-old male with chronic complete tetraplegia who presented to the emergency room with unremitting autonomic dysreflexia (AD). He was admitted for medical management of the AD when a source of the AD could not be identified. After an extensive workup, the patient was found to have bilateral sacral insufficiency fractures. He subsequently underwent sacroplasty with immediate and full resolution of AD symptoms and return to full premorbid function within 24 h.

**Discussion:**

People with chronic spinal cord injury (SCI) are at risk for spinal column fragility fractures in addition to lower extremity fractures. Vertebroplasty may be a safe option for treatment of insufficiency fractures in situations where conservative care may put people at risk for significant immobility-associated complications.

## Introduction

Bone mineral density loss and its associated fractures can lead to significant complications and morbidity for individuals with spinal cord injury (SCI). Fractures typically occur in the extremities below the level of the lesion, most commonly in the distal femur and proximal tibia [1]. In contrast, pathologic fractures of the spinal column have not been reported in the literature and are not considered a common risk for people with SCI. Treatment of fragility fractures among people with SCI is typically conservative and involves mobility restrictions, often associated with a decline in functional independence and an increase in burden of care. We present for the first time a case of pathologic bilateral sacral insufficiency fractures causing intractable autonomic dysreflexia (AD) and loss of mobility and function which was collaboratively identified and aggressively managed by the Hospitalist, SCI Physiatry, and Interventional Radiology to allow for rapid return to function with resolution of AD.

## Case presentation

A 52-year-old male with history of C6 American Spinal Injury Association Impairment Scale (AIS) A spinal cord injury sustained during a bicycle accident in 1993 presented to the emergency department of a community hospital with AD characterized by hypertension >190/100, diaphoresis, clamminess in the extremities and nonspecific abdominal discomfort. The patient’s relevant medical history includes a stage IV sacral wound, complicated by perirectal fistula and osteomyelitis requiring surgical debridement with partial ostectomy and myocutaneous flap closure ~1 year prior to this presentation. Additionally, the patient utilizes clean intermittent catheterization for management of neurogenic bladder and has a history of multiple urinary tract infections (UTIs). The patient’s baseline functional status includes mobility at a power wheelchair level with bilateral tenodesis splints to assist with hand function. He is gainfully employed full time.

The patient was admitted to the hospital because the source of the AD could not be identified. Initial infectious workup including bloodwork and urine sample was negative. Upon admission, the patient’s Hospitalist consulted Gastroenterology and SCI Physiatry from an early stage; the ensuing workup and treatment of this individual was a collaborative team approach. Abdominal etiology of the AD was suspected, and a CT scan of the abdomen showed partial bowel obstruction, for which treatment included restriction of oral feeding and a nasogastric tube for decompression. A repeat abdominal CT scan showed resolution of the obstruction and his diet was advanced and the nasogastric tube was removed. Because the AD persisted, further workup ensued and included esophagogastroduodenoscopy (EGD), small bowel follow through, and HIDA scan, which were all unremarkable.

During this time, the patient noticed that AD symptoms seemed to be positional; associated with sitting upright in his wheelchair and relieved with supine position in the bed. Positional assessment of blood pressure (BP) was performed and supine BP was 128/91, increasing to 175/114 after transfer to his wheelchair. Due to the patient’s history of rectal fistula and flap procedure and the onset of AD associated with sitting in his wheelchair, the Hospitalist and Physiatrist collaboratively determined the next step to be MRI of the pelvis. This showed bilateral nondisplaced sacral insufficiency fractures with midline transverse component across S2 with associated bone marrow edema.

Initial Orthopaedic Surgery evaluation resulted in a recommendation to defer surgical intervention in favour of a conservative approach to treatment consisting of bedrest for 4–6 weeks due to concern for complications associated with invasive procedures in people with chronic complete tetraplegia. However, it was felt that the risk of potentially prolonged bedrest in this individual with chronic tetraplegia carried a significant risk of continued AD, skin breakdown, thromboembolism, and delayed fracture healing, and for that reason the team consulted Interventional Radiology. The following day the patient underwent sacroplasty without complications. Immediately following sacroplasty, the patient was able to sit in the wheelchair for a prolonged period with no episodes of AD and he was discharged home the same day of the procedure. The patient returned to work the following day. CT scan of the abdomen and pelvis at 2 weeks post-operatively showed cement in the sacral alae with no evidence of nonunion. At a 3-month follow-up with the patient’s Primary Care Physician, the patient had no complications from the surgery.

## Discussion

Here we describe the novel presentation and treatment of bilateral pathologic sacral insufficiency fractures causing AD in an individual with chronic tetraplegia with sacroplasty resulting in immediate resolution of AD and return to premorbid function without complication. This case exemplifies the importance of active collaboration amongst the Patient-Hospitalist-Specialist(s) team to achieve excellent outcomes in the care of complicated patients with SCI.

A previous case report by Kanezaki et al. discussed an individual with chronic paraplegia who had nondisplaced bilateral nonunion of the sacrum hypothesized to be secondary to acute fractures of the bilateral sacral ala after a fall from standing. In that case, the patient presented with low back pain refractory to analgesia and immobilization leading to 8 months of reduced mobility. The patient ultimately underwent trans-sacral bar osteosynthesis with spinopelvic fixation with rehabilitation and weight bearing exercises after surgery. One-year follow-up CT scan showed complete union of the bilateral sacral ala [2]. The case we present differs, in that the presentation was nonspecific (AD), the fractures were unrelated to trauma, and the interventional approach resulted in immediate resolution of life-threatening symptoms and no loss of functional abilities.

The rate of bone loss in individuals with motor complete SCI is estimated at 1% of bone mineral density per week in the first 6 months after injury and continues to be heightened for the next 3–8 years [1]. Reiter et al. showed continued active resorption of bone in individuals with SCI of more than 5 years [3]. The rate of bone loss in individuals with SCI surpasses that of individuals on bed rest or postmenopausal women not on antiresorptive medications, and the mechanisms underlying this increased rate of bone loss are complex and not fully understood, although it appears to be associated with both increased osteoclastic and decreased osteoblastic activity [1, 4].

The rate of fractures in individuals with SCI are 1.2–3.4 per 100 patient years, and Logan et al. showed an incidence of at least one fracture below the level of injury to be 33% at 21-year follow-up [4, 5]. Risk factors for osteoporosis and fracture in individuals with SCI include motor complete SCI, lower level of injury, longer duration of injury, increased alcohol consumption, anticonvulsant medication use, lower body mass index, increasing age, past history of fracture, and female sex. Additionally, individuals with SCI are at risk for low-impact fractures, which may occur during transfers or positional changes in bed [1, 4]. Although the occurrence of lower body long bone fractures in individuals with chronic SCI has been well documented, in this case we present a patient with fragility fractures to the sacrum. This became the source of dangerous episodes of intractable AD which led to a prolonged hospital course for the patient. Pathologic spinal column fractures secondary to osteoporosis must be considered in this patient population, especially as people with SCI continue to experience greater longevity with SCI.

Further complicating the presentation, the perception of a fracture may be altered due to impaired sensation, and signs/symptoms may be subtle, such as increased spasticity, minimal or no swelling, or, as in this case, unexplained episodes of AD [1]. Management is often conservative, putting individuals with SCI at increased risk for complications associated with prolonged bed rest including pressure injuries, bowel dysfunction, deep vein thrombosis, contractures, and respiratory dysfunction [6]. In this case, the complications associated with prolonged bedrest were avoided with immediate resolution of AD after sacroplasty without surgical complications. As the treatment of individuals with SCI improves, life expectancy increases, and opportunities for improved mobility are created, minimally invasive options should be considered in the management of fractures.

Vertebroplasty is the injection of polymethylcrylate into a fractured vertebral body and is commonly used in the general population for the treatment of symptomatic osteoporotic fractures. Clark et al. showed a significant decrease in Numeric Rating Scale for pain in patients who underwent vertebroplasty over a control group from 14 days to 6 months post-procedure. This study is significant in that 57% of the patients included were hospitalized patients, which indicates a higher level of disability at the time of vertebroplasty. Median duration of hospital stay was 5.5 days shorter for the hospitalized patients in the vertebroplasty group versus controls [7]. The safety and efficacy of this procedure, which has been proven in debilitated patients, was reiterated in our patient’s case. Despite hospitalization for over 1 week, vertebroplasty facilitated a return to his premorbid functional status and same day discharge from inpatient hospitalization.

The multidisciplinary approach between the patient, primary care (Hospitalist), and specialists (SCI Physiatrist, Gastroenterologist, Interventional Radiologist) was integral to the positive outcome for this patient. The SCI Physiatrist who had followed the patient since the time of his injury was able to provide significant insight into the previous complications experienced by the patient and his baseline functional status. After discovery of the positional impact on the episodes of AD by the patient and the Hospitalist, imaging was obtained which led to the final diagnosis. Additionally, the knowledge of the patient’s baseline high level of function ultimately impacted the decision to undergo sacroplasty and avoid prolonged bedrest. Multidisciplinary discussions regarding the risks and benefits of each treatment option led to a successful outcome for this individual. We advocate for clinicians to actively seek out communication amongst multiple health care providers in both the inpatient and outpatient settings to best meet the complex health care needs of people with SCI.

Pathologic fractures secondary to osteoporosis occur in chronic SCI patients not only in the long bones of the lower extremity but also the spinal column and should be considered in a clinician’s differential diagnosis. Immediate resolution of AD and prevention of complications associated with prolonged bed rest with quick return to prior functional status are significant indications for the use of minimally invasive procedures for the treatment of spinal column fragility fractures in the SCI population. Multidisciplinary collaboration of health care providers is integral to positive outcomes for patients with SCI.
